# Bacterial diversity associated with the abdomens of naturally *Plasmodium*-infected and non-infected *Nyssorhynchus darlingi*

**DOI:** 10.1186/s12866-020-01861-0

**Published:** 2020-06-25

**Authors:** Tatiane Marques Porangaba Oliveira, Sabri Saeed Sanabani, Maria Anice Mureb Sallum

**Affiliations:** 1 Departamento de Epidemiologia, Faculdade de Saúde Pública, Universidade de São Paulo, São Paulo, SP Brazil; 2 LIM-3, Hospital das Clínicas da FMUSP (HCFMUSP), Faculty of Medicine, University of São Paulo, São Paulo, Brazil

**Keywords:** *Pseudomonas*, *Escherichia*/*Shigella*, Malaria, *Ny. darlingi*, Amazon, *Plasmodium*

## Abstract

**Background:**

The bacterial community present in the abdomen in Anophelinae mosquitoes can influence mosquito susceptibility to *Plasmodium* infection. Little is known about the bacteria associated with *Nyssorhynchus darlingi*, a primary malaria vector in the Amazon basin. We investigated the abdominal bacterial community compositions of naturally *Plasmodium*-infected (*P*-positive, *n* = 9) and non-infected (*P*-negative, *n* = 7) *Ny. darlingi* from the Brazilian Amazon region through massive parallel sequencing of the bacterial V4 variable region of the 16S rRNA gene.

**Results:**

Bacterial richness of *Ny. darlingi* encompassed 379 operational taxonomic units (OTUs), the majority of them belonging to the *Proteobacteria*, *Firmicutes* and *Bacteroides* phyla. *Escherichia*/*Shigella* and *Pseudomonas* were more abundant in the *P*-positive and *P*-negative groups, respectively, than in the opposite groups. *Enterobacter* was found only in the *P*-negative group. The results of statistical analyses conducted to compare bacterial abundance and diversity between *Plasmodium*-infected and *Plasmodium*-non-infected mosquitoes were not significant.

**Conclusions:**

This study increased knowledge about bacterial composition in *Ny. darlingi* and revealed that *Plasmodium*-positive and *Plasmodium*-negative groups share a common core of bacteria. The genera *Prevotella* 9, *Sphingomonas*, *Bacteroides*, and *Bacillus* were reported for the first time in *Ny. darlingi.*

## Background

Malaria is one of the world’s most common and deadly tropical diseases. Recent data from the World Health Organization (WHO) has estimated that there were 219 million clinical cases and 435,000 estimated deaths in 87 malaria-endemic countries with ongoing malaria transmission in 2017 [1]. Brazil, Nicaragua and Venezuela registered increased malaria incidences in 2017. Malaria infections in Brazil are predominantly caused by *Plasmodium vivax*, accounting for > 84% of cases [2]. Most malaria cases occur in the Amazon region, where the primary vector is *Nyssorhynchus darlingi*.

It is known that the mosquito midgut microbiota can affect the development of *Plasmodium* parasites in mosquitoes [3–8]. Isolates of *Enterobacter amnigenus* and *Enterobacter cloacae* are able to impair the sporogonic development of *P. vivax,* while *Serratia marcescens* completely inhibits *P. vivax* oocyst development in *Anopheles albimanus* [9]. One *Enterobacter* sp. strain (*Esp_Z*) isolated from the gut of wild *Anopheles arabiensis* confers resistance to *P. falciparum* infection in *Anopheles gambiae* [3]*.* Other studies have reported that *Serratia marcescens* blocks the development of *P. falciparum* ookinetes in *An. gambiae* [4, 10]. It has been proposed that bacteria present in the midgut modulate *Plasmodium* infection in the mosquito through at least two mechanisms: production of bacterial metabolites that impair the development of the parasite and induction of the immune response [3, 10–12].

Following an infective blood meal, the numbers of ookinetes present in the midgut lumens of aseptic and septic *An. gambiae* are similar, while the numbers of ookinetes present in the midgut epithelia of aseptic mosquitoes are 2.5 times higher than those in septic mosquitoes, suggesting that the bacterial effect occurs during ookinete development and invasion of the midgut epithelium [11]. Additionally, *Anopheles* and *Aedes* mosquitoes that are fed antibiotics to reduce microbiota populations have higher rates of *Plasmodium* and dengue virus infection than untreated mosquitoes [13]. Thus, symbiotic bacteria can be an alternative tool for blocking *Plasmodium* development in mosquitoes by decreasing vector competence.

The interaction between insects and microbes has been studied in a variety of vector species; however, little is known about the bacterial communities associated with the abdomen in the primary neotropical vector, *Ny. darlingi* [14–16]. This study compared the bacterial communities associated with the abdomens of non-infected and *Plasmodium-*infected field-collected females of *Ny. darlingi.*

## Results

### Sequencing data output

Twenty-four female abdomens were employed to generate bacterial community data. Sixteen samples (66.6%) were successfully amplified and sequenced for the 250 bp 16S rRNA genomic region. Nine were infected with *Plasmodium* (3 from Cruzeiro do Sul, 1 from Mâncio Lima, 1 from Lábrea, and 4 from Machadinho D’Oeste), and seven were not infected with *Plasmodium* (6 from Cruzeiro do Sul and 1 from Machadinho D’Oeste) (Additional file 1).

The MiSeq Illumina platform generated 2,505,232 raw reads (R1 and R2) from 16 abdomens, with a median of 135,829 reads (range: 28,430 – 434,102). The read values were 1,379,570 for the *P*-positive samples and 1,125,662 for the *P*-negative samples. After assembly and filtering of the OTUs with less than 5 sequences, 275,203 sequences were retained. These sequences were assigned to 407 unique OTUs (Additional file 2); 96 were found only in the *P*-positive group, 172 were found only in the *P*-negative group, and 139 were identified in both groups.

### Bacterial compositions

Twenty-eight OTUs corresponded to chloroplasts, mitochondria, and unassigned OTUs. Of the 379 bacterial OTUs obtained, 333 were identified to the genus level. Additionally, 35 OTUs could only be identified at the family level, 4 OTUs could only be identified at the order level, 6 could only be identified at the class level, and one could only be identified at the phylum level.

**Fig. 1:**
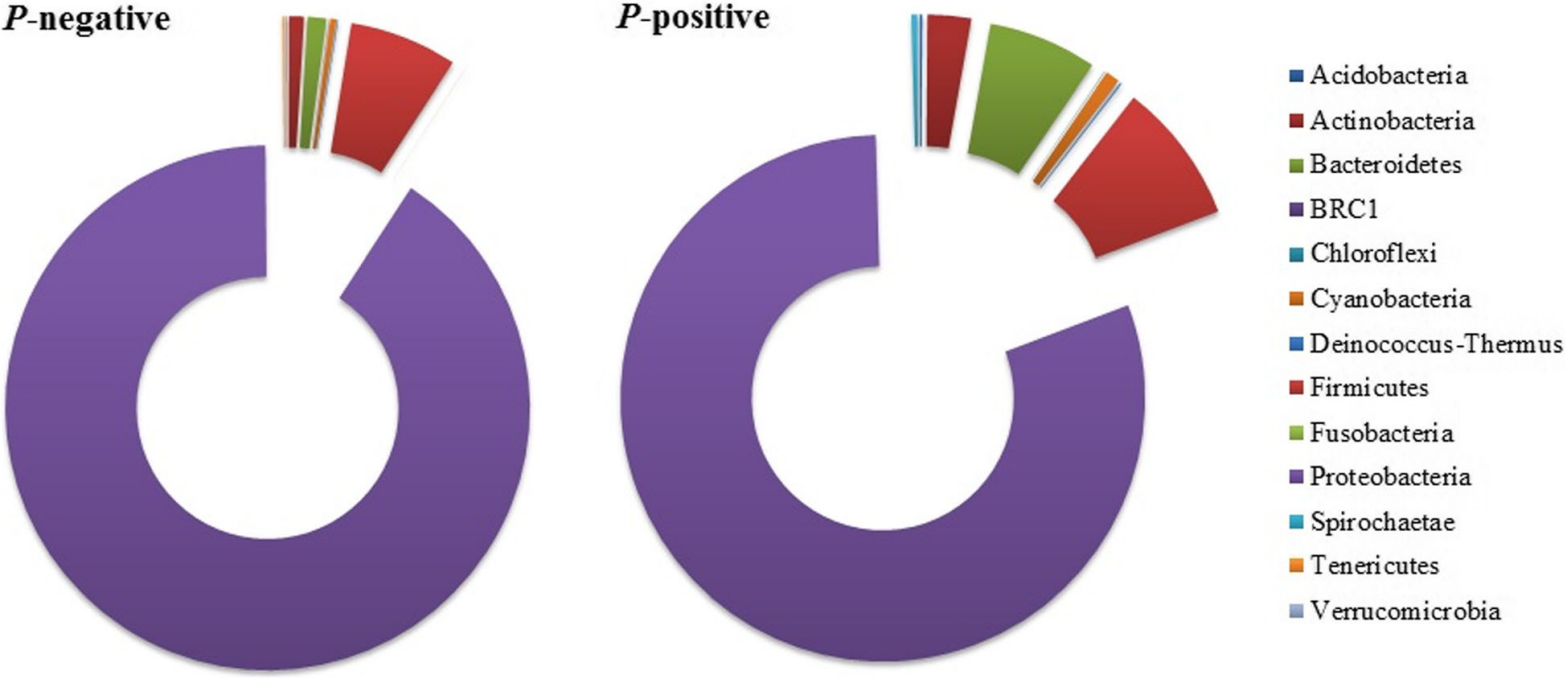
Composition of bacterial phyla in the abdomen of *P*-negative and *P*-positive groups *Nyssorhynchus darlingi* after filtering non-bacterial sequences

After filtering of the non-bacterial sequences, the sequences detected in the *P*-negative group were assigned to 294 bacterial OTUs and sorted into 12 phyla, among which *Proteobacteria* (90.1%), *Firmicutes* (6.6%) and *Bacteroidetes* (1.15%) were the most abundant and accounted for 98% of the sequence reads. The most abundant bacterial phyla of the 214 bacterial OTUs within the *P*-positive group were *Proteobacteria* (80.3%), *Firmicutes* (8.7%), *Bacteroidetes* (6.7%) and *Actinobacteria* (2.7%) (Fig. 1). *Gammaproteobacteria* was the predominant class in both groups, but its families had different tendencies. Within the phylum *Proteobacteria*, *Pseudomonadaceae* was predominant in the *P*-negative samples, while *Enterobacteriaceae* was predominant in the *P*-positive samples.

A Venn diagram was employed to display the common and unique bacterial OTUs observed at the genus level in both groups (Additional file 3). In the *P*-negative group, 294 bacterial OTUs were detected, while in the *P*-positive group, 214 bacterial OTUs were detected. The proportion of unique bacterial OTUs was 43.54% in the *P*-negative group and 22.43% in the *P*-positive group. The percentage of bacterial OTUs detected in both groups was 34.04%.

**Fig. 2:**
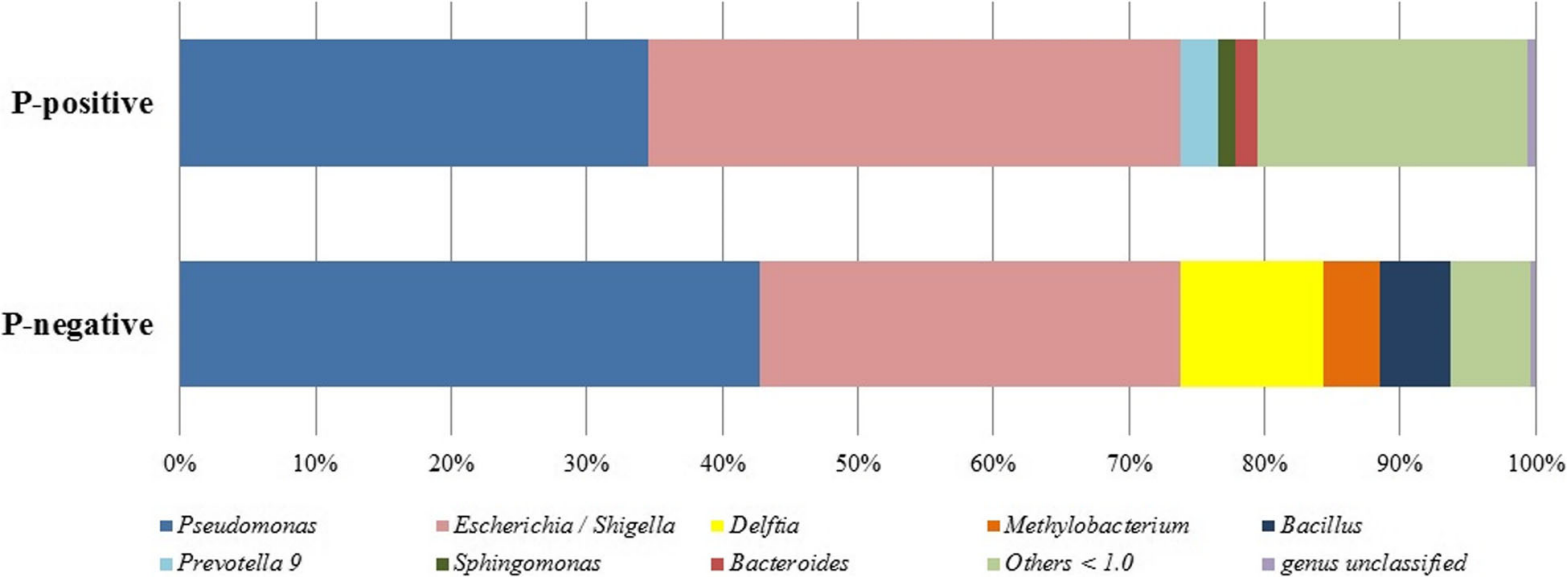
Abundance of the bacterial genera in *P*-positive and *P*-negative *Nyssorhynchus darlingi* after filtering non-bacterial sequences. Only genera that had a relative abundance of 1% or greater are presented. Others < 1.0 corresponds to genera that had relative abundance < 1%

The genera *Pseudomonas* and *Escherichia*/*Shigella* were the most abundant in the *P*-negative and *P*-positive groups, respectively, compared with the opposite groups. Considering the genera that had relative abundances of more than 1%, *Prevotella* 9, *Sphingomonas* and *Bacteroides* were found in the *P*-positive group, and *Delftia*, *Methylobacterium* and *Bacillus* were found in the *P*-negative group (Fig. 2). According to the results of previous studies on the microbiota of Anophelinae mosquitoes, the genera *Prevotella* 9, *Sphingomonas*, *Bacteroides* and *Bacillus* have never been associated with the abdomen of *Ny. darlingi*. Composition of the bacterial OTUs from each sample can be viewed in Additional file 4.

### PCoA and alpha and beta diversity analyses

A rarefaction curve was created with a sampling depth of 1500 sequences, showing that the sequencing depth was adequate to infer the structure and abundance of the bacterial community in the abdomen of *Ny. darlingi* (Additional file 5). Two sequenced samples (AC141–7 and AC144–17) had sequence numbers lower than the depth used for the rarefaction and therefore were not used in the diversity analyses. To address the bacterial diversity, we used Shannon’s diversity index, also known as the Shannon-Weaver index, which considers the richness and relative abundance of OTUs. The results showed no significant difference between the *P-*negative (2.78 ± 0.71) and *P-*positive (2.68 ± 1.24) groups (Additional file 6).

**Fig. 3:**
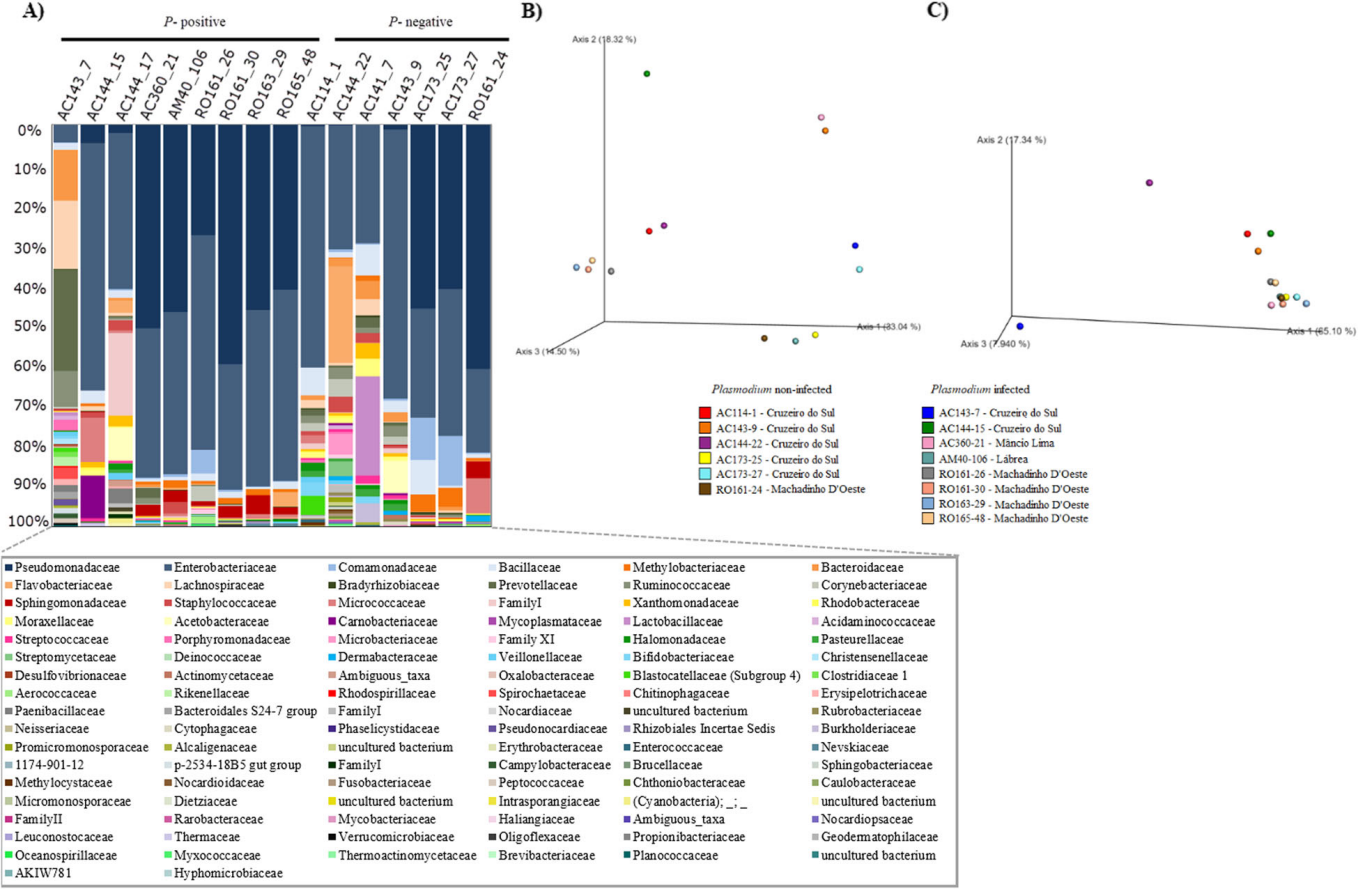
Beta diversity. Composition of OTUs at family level of all 16 samples (**a**) and Principal Coordinates Analysis based on the unweighted (**b**) and weighted (**c**) UniFrac distances

The beta diversity distances between both groups were measured using PCoA. Both PCoA plots of unweighted and weighted UniFrac distances not defined clustering of *P*-negative versus *P*-positive samples (Fig. 3). The PERMANOVA analysis did not detect any difference in the bacterial composition between the *P*-positive and *P*-negative groups (*p* > 0.05). For the above analyses, data from the 407 OTUs generated were used.

Because no significant difference was found in bacterial composition when comparing infected and non-infected groups when samples from all localities were included, beta diversity (PERMANOVA) analysis was performed to compare infected and non-infected groups encompassing females collected in Cruzeiro do Sul and Machadinho D’Oeste separately. The analysis was performed between (1) the infected and non-infected groups (infected = 3; non-infected = 6) from Cruzeiro do Sul and (2) the infected and non-infected groups (infected = 4; non-infected = 1) from Machadinho D’Oeste. In both PERMANOVA analyses, there was no significant difference between the groups analysed (Cruzeiro do Sul, *p* = 0.681; Machadinho D’Oeste, *p* = 0.373).

## Discussion

In this study, the bacterial community associated with the abdomen of *Ny. darlingi* was investigated. In addition, the bacterial diversity was compared between the two groups studied (the *P*-negative and *P*-positive groups). Next-generation sequencing of PCR products was used to identify the bacterial community associated with the female abdomen. The DNA samples from 7 non-engorged mosquitoes failed to exhibit amplification of 16S rRNA after several trials, likely because of the presence of PCR inhibitors or the low numbers of bacteria in these samples. This result is corroborated by those of other studies, such as an investigation conducted by Terenius et al. [14], in which only one of four non-blood-fed *Ny. darlingi* presented amplification of the16S rRNA gene.

The bacteria in adult *Ny. darlingi* abdomens comprised three predominant phyla: *Proteobacteria*, *Firmicutes* and *Bacteroidetes*. This result presents similarity to the results of other studies performed with Anophelinae mosquitoes [17, 18] and suggests that bacteria of these phyla have importance in the physiology of Anophelinae mosquitoes. The number of OTUs identified was higher than those found in other conventional molecular studies [14]. Of the 379 bacterial OTUs, only 7 showed an abundance higher than 2%, suggesting that there are few predominant bacterial genera. Minard et al. [19] reported that mosquito females are colonized mainly by *Gammaproteobacteria*. Similarly, *Gammaproteobacteria* was the most prevalent class of bacteria identified in the females analysed (16/16 samples). *Gammaproteobacteria* includes the *Enterobacteriaceae* and *Pseudomonadaceae* families.

*Enterobacteriaceae* has been found in abundance in *Plasmodium*-infected *An. gambiae*, indicating that bacteria of this family contribute to the development of the parasite [20]. Although *Enterobacteriaceae* was the most abundant family identified in the *Ny. darlingi P*-positive group, there was no significant difference in diversity between the *P*-positive and *P*-negative groups (*p* > 0.05). Inconsistent results of studies can be caused by various factors, including differences in experimental design and sample preparation. For example, the females used by Boissière et al. [20] were fed *P. falciparum*-infected blood, and the midgut microbiota was analysed 8 days after the blood meal. In our study, field-collected females were found naturally infected with *Plasmodium,* but we did not have information about the time of the mosquito blood meal. Considering that the females were naturally infected, they would have fed on *Plasmodium*-infected human blood at least 10–15 days before [21]. The differences observed may be associated with the time of the infected blood meal and the HLC collection procedure.

*Anopheles gambiae* exposed to an *Enterobacter* (*Esp_Z*) isolate have been found to become less susceptible to *P. falciparum* infection [3]. *Enterobacter* was recovered from 2 *Ny. darlingi* females, neither of which was infected with *Plasmodium*. Despite this finding, it is premature to hypothesize that females with *Enterobacter* are less susceptible to *P. falciparum* infection than those without it, because our sample was small and limited to a few localities with moderate, low or moderately high transmission [22]. These two limitations prevent us from drawing any conclusions about the impact of *Enterobacter* on vector species susceptibility to *P. falciparum* infection. Further studies will be necessary to verify whether any *Enterobacter* isolates can decrease *Ny. darlingi* susceptibility to *Plasmodium* infection and to determine if the inhibition mechanism is similar to that of the *Esp_Z* isolate in *An. gambiae*, which is mediated by the generation of reactive oxygen species (ROS).

*Pseudomonas* was found in *Ny. darlingi* and has been widely reported in anopheline mosquitoes from Africa, Asia and America [14, 15, 23–25]. In this study, *Pseudomonas* was the most abundant genus in the *P*-negative group and was detected in 15 out of 16 samples analysed. These data corroborate those of a study by Chavshin et al. [26], who reported *Pseudomonas* as the most common bacterial isolate in *Anopheles stephensi* larvae and adults, indicating possible transmission of *Pseudomonas* between mosquito developmental stages. The high prevalence of *Pseudomonas* in *Anopheles* mosquitoes suggests the capability of *Pseudomonas* to easily adapt to the midgut environment.

The most predominant bacteria in the *P*-positive group detected in all investigated samples were found to belong to the genus *Escherichia*/*Shigella*. In previous studies, this genus has been found in all mosquito samples analysed in Northern California (USA) [27] and in more than 85% of samples of *An. gambiae* [20]. Both studies have also reported the prevalence of this genus in other species of mosquitoes. The fact that these bacteria were found in *Ny. darlingi* breeding sites located in Manaus in the Brazilian Amazon region [28] suggests that this genus is either acquired during immature development or immediately after emergence.

Although the *P*-negative group had a higher number of OTUs than the *P*-positive group, there was no significant difference in bacterial diversity between the groups (*P*-positive and *P*-negative); neither group was more diverse than the other. Additionally, there was no significant difference in the composition of the bacteria between the groups (beta diversity), not even between the infected and non-infected groups of the same municipality (Cruzeiro do Sul and Machadinho D’Oeste). These results appear contrary to the findings of Bassene et al. [29], who reported greater bacterial diversity in vectors (*An. gambiae* and *An. funestus*) infected with *P. falciparum*. Possibly, this difference in results could be related to the fact that we did not differentiate whether the *Ny. darlingi* infection was caused by *P. vivax* or *P. falciparum.*

Another possible explanation for the lack of statistical significance difference, not addressed here, could be due to the effects of *Ny. darlingi* genotype variant on microbial communities [30–32]. Several studies have shown that various factors such as nutrition, sex, environment, and genotype can all shape the pattern of mosquitoes-associated microbiota [19, 33]. Despite these reports, Minard et al. [33] demonstrated that the *Aedes albopictus* genetic variation is correlated with microbiome composition. However, in another study with populations of *Ae. albopictus* reared in the laboratory, Minard et al. [34] verified that when environmental factors were removed, there were no differences in the microbiota diversity between the populations studied. Dickson et al. [35] found that the microbiota diversity in the *Aedes aegypti* is determined by the environment, regardless of the host genotype. Thus, further studies are needed to verify whether different genotypes of *Ny. darlingi* can influence the bacterial composition of the microbiota.

Considering that our study employed field collected females, the number of specimens analyzed was small. Further studies will be needed to test the hypotheses and address if the load of *Plasmodium* parasites is associated with the divergence in the mosquito gut microbiota.

## Conclusions

Here, we provide data on bacterial communities associated with the abdomens of *Ny. darlingi* naturally infected and not infected with *Plasmodium*. The genera *Prevotella* 9, *Sphingomonas*, *Bacteroides* and *Bacillus* are reported for the first time in *Ny. darlingi*. Our data contribute to a better understanding of the bacteriome composition in *Ny. darlingi*. Since *Enterobacter* was found in two non-infected *Ny. darlingi* samples and was absent in the *P*-positive group, other studies are necessary to determine if *Enterobacter* species present in *Ny. darlingi* offer protection against *Plasmodium* infection.

## Methods

### Mosquito collections

Females of *Ny. darlingi* were collected in the municipalities of Cruzeiro do Sul (Acre state), Mâncio Lima (Acre state), Lábrea (Amazonas state), and Machadinho D’Oeste (Rondônia state), Brazilian Amazon (Additional file 1). Field collections were conducted in peridomestic habitats by human landing catches (HLCs). Mosquitoes were killed with ethyl acetate (C_4_H_8_O_2_) and immediately preserved in silica gel until species identification. Mosquito species were identified using morphological characteristics. Following identification, females were preserved at − 80 °C.

### Plasmodium testing

Each *Ny. darlingi* female collected was divided into the head/thorax and abdomen. The DNA of the head/thorax was extracted according to Laporta et al. [36] and used for *Plasmodium* testing. *Plasmodium* detection was performed by using real-time PCR of the 18S rRNA region followed by high-resolution melting (HRM) analysis. For each 20 μl reaction, 1X MeltDoctor™ HRM Master Mix (ThermoFisher Scientific, Inc., Waltham, MA, USA), 10 ng of DNA, 500 nM of each primer (1459-M13[forward, 5’TGTAAAACGACGGCCAGTCTGGTTAATTCCGATAAC 3′] and 1706-M13 [reverse, 5’CAGGAAACAGCTATGACCTAAACTTCCTTGTGTTAGAC 3′]) [37] and commercial ultrapure H_2_O were added. The cycling program consisted of 95 °C for 10 min followed by 50 cycles of 95 °C for 15 s and 60 °C for 1 min. The reaction was then followed by HRM curve analysis, which was conducted by increasing the temperature from 60 °C to 95 °C with a ramp rate of 0.3%. The results were analysed using StepOnePlus™ Software v2.3 and HRM v3.0.1 Software. Seventy samples were infected with *Plasmodium*. The corresponding abdomens from 13 infected samples and 11 *Plasmodium*-uninfected female *Ny. darlingi* were used for the 16S rRNA amplicon survey (Additional file 1).

### Sequencing of the V4 region of 16S rRNA

Abdomens of *Ny. darlingi* were surface rinsed twice in 70% ethanol and ultrapure water. The genomic DNA of each individual abdomen was extracted by using a DNeasy PowerSoil kit (Qiagen, Hilden, Germany) or by the salt precipitation method [36]. For the DNeasy PowerSoil Kit, each abdomen was placed in PowerBead tubes (included in the PowerSoil kit) and incubated at room temperature for 15 min before bead beating for another 15 min. After these steps, genomic DNA was extracted according to the manufacturer’s instructions. For the salt precipitation method, each abdomen was mixed in 500 μL of TEN buffer (2 mM Tris-HCl pH 8.0, 0.5 mM EDTA, 5 mM NaCl) and homogenized using 1-mm-diameter zirconia beads (Biospec, Bartlesville, USA) via the use of BeadBlaster24 equipment (Benchmark Scientific, Inc., Sayreville, NJ, USA) for 4 cycles with shaking (4 m/sec) for 40 s followed by 20 s without shaking in each cycle. A lysis buffer containing 5 μL of 10% SDS (Promega, Madison, USA) and 3 μL of 20 mg/mL proteinase K (Promega) was added to the homogenate and mixed. After 1 h of incubation at 56 °C, 150 μL of 5 M NaCl was added, and the mixture was shaken vigorously for 15 s. The mixture was centrifuged for 10 min at 13,000 rpm at 20 °C, and the supernatant was gently transferred into a new tube. Immediately, 600 μL of freshly prepared cold isopropyl alcohol was added to the supernatant and mixed by inversion, and the mixture was incubated at − 20 °C for 48 h. The supernatant was decanted after a 10 min centrifugation. The remaining pellet was washed with 1 mL of 70% ethanol, and the dried pellet was resuspended in 20 μL of TE buffer (2 mM Tris-HCl, pH 8.0, 0.5 mM EDTA).

The V4 hypervariable region of the 16S rRNA gene was amplified according to Caporaso et al. [38]. Briefly, each reaction was performed in a final volume of 20 μL consisting of 1X GoTaq® Colorless Master Mix (Promega), 0.3 μM of each primer (Additional file 7), 2 μL of genomic DNA and ultrapure water. The thermocycling conditions were 94 °C for 3 min followed by 29 cycles of 94 °C for 45 s, 50 °C for 1 min, 72 °C for 1 min and 30 s, and a final extension of 72 °C for 10 min. The reactions were carried out in triplicate. The PCR products were visualized on a 2% agarose gel stained with Gelred® (Uniscience, Miami, USA). The PCR products were purified with Agencourt AMPure XP magnetic beads (Beckman Coulter, Brea, USA) and then quantified by real-time PCR with KAPA (KAPA Biosystems, Wilmington, USA) according to the manufacturer’s recommendations.

All samples were normalized to 3 nM, and an equimolar pool of DNA was prepared. Next-generation sequencing of the V4 region was performed on an Illumina MiSeq sequencer (Illumina, San Diego, USA) using a MiSeq Reagent Micro v2 kit (300 cycles).

### Processing of sequences and taxonomic attribution

Illumina paired-end reads were assembled in QIIME v.1.9 [39] with a minimum overlap of 20 base pairs (*join_paired_ends.py*). The sequences were filtered based on the length and sequencing error rates (E_max = 1). USEARCH v.11 [40, 41] was used to filter sequences (*fastq_filter*), discard singletons (*sortbysize*), and remove chimaeras and cluster sequences in unique OTUs (*cluster_otus*). The taxonomic classification of each read was assigned against Silva v.128 [42] at a 99% threshold of pairwise sequence similarity using QIIME v.1.9. All OTUs that had fewer than 5 sequences were removed from the analysis.

### Diversity index determination and statistical analysis

The Shannon-Weaver index (H) and observed OTU index (S) were used to describe the bacterial diversity (alpha diversity) of each separate specimen and each group (*P*-positive and *P*-negative). The Wilcoxon-Mann-Whitney test was used to compare the results for both groups. Dissimilarity analyses (beta-diversity) between the *P*-positive and *P*-negative groups were performed using the unweighted and weighted UniFrac distances and a sampling depth of 1500 sequences. The unweighted UniFrac distance is a qualitative measure that uses phylogenetic information to compare biological communities, while the weighted Unifrac distance is a quantitative measure. Principal coordinate analyses (PCoA) of the unweighted and weighted UniFrac distances of bacterial communities were conducted to measure the distance between communities. Permutational multivariate analysis of variance (PERMANOVA) was employed to measure intergroup distances. The PCoA and PERMANOVA analyses were conducted with rarefied data recovered after rarefying steps in QIIME2 (2019.1 version).

## Supplementary information

**Additional file 1. **ID sample, Municipality of collection and *Plasmodium* infection of each sample used in this study

**Additional file 2.** Number of OTUs sequences from each sample, after removing OTUs with less than 5 sequences.

**Additional file 3. **Venn diagram display the number of unique bacterial OTUs in *P*-negative and *P*-positive *Ny. darlingi*. Number of OTUs after filtering non-bacterial sequences.

**Additional file 4.** Composition of abundant OTUs at family level of all 16 samples after filtering non-bacterial sequences. Samples collected in Cruzeiro do Sul: AC114–1, AC141–7, AC143–9, AC173–25, AC173–27, AC143–7, AC144–22, AC144–15 and AC144–17. Sample collected in Mâncio Lima: AC360–21. Sample collected in Lábrea: AM40–106. Samples collected in Machadinho D’Oeste: RO161–24, RO161–26, RO161–30, RO163–29 and RO165–48.

**Additional file 5.** Rarefaction curves of OTUs (97% similarity) of V4 region of 16S rRNA gene sequences from 16 samples. Rarefaction curves were generated in Qiime2 (2019.1 version). Bars correspond to the standard deviation in each depth step after 10 iterations (rarefied tables computed at each sampling depth).

**Additional file 6 **Box plot of Shannon index for *P*-negative and *P*-positive groups. Center lines show the medians; box limits indicate the 25th and 75th percentiles; whiskers extend to 5th and 95th percentiles, outliers are represented by dots.

**Additional file 7.** Sequence of the oligonucleotides used to amplify the V4 region of the 16S rRNA gene.

## Data Availability

The datasets generated and analysed during the current study are available in the European Nucleotide Archive (ENA) repository (Project: PRJEB32570, Access numbers: ERR3324411-ERR3324396). The sequences of the 407 OTUs are available in the Zenodo repository (10.5281/zenodo.3738681).

## Abbreviations

DNA: Deoxyribonucleic Acid
EDTA: Ethylenediamine Tetraacetic Acid
HCL: Hydrochloric Acid
HLC: Human Landing Catch
HRM: High-Resolution Melting
NaCl: Sodium Chloride
OTU: Operational Taxonomic Unit
PCoA: Principal Coordinates Analysis
PCR: Polymerase Chain Reaction
PERMANOVA: Permutational Multivariate Analysis of Variance
QIIME: Quantitative Insights Into Microbial Ecology
rRNA: Ribosomal Ribonucleic Acid
ROS: Reactive Oxygen Species
SDS: Sodium Dodecyl Sulfate
TE: Tris-EDTA
WHO: World Health Organization
