# Supplementary material for: Bacterial diversity associated with the abdomens of naturally Plasmodium-infected and non-infected Nyssorhynchus darlingi
Source: BMC Microbiol. 2020 Jun 25;20:180. doi: 10.1186/s12866-020-01861-0 (PMC7315559; doi:10.1186/s12866-020-01861-0)
Supplement: Supplementary file 1 — Additional file 1. ID sample, Municipality of collection and Plasmodium infection of each sample used in this study [file 12866_2020_1861_MOESM1_ESM.docx]

**Supplementary information**

**Bacterial diversity associated with the abdomen of naturally *Plasmodium*-infected and non-infected *Nyssorhynchus darlingi***

Tatiane M. P. Oliveira, Sabri S. Sanabani, Maria Anice M. Sallum

**Additional file 1**. ID sample, Municipality of collection and *Plasmodium* infection of each sample used in this study.

| ID sample | Municipality | Collection date | *Plasmodium* infection | DNA extraction method |
| --- | --- | --- | --- | --- |
| AC114-1 | Cruzeiro do Sul | 05/11/2015 | Non-infected | Commercial kit |
| AC141-2* | Cruzeiro do Sul | 04/20/2015 | Non-infected | Commercial kit |
| AC141-5* | Cruzeiro do Sul | 04/20/2015 | Infected | Commercial kit |
| AC141-6* | Cruzeiro do Sul | 04/20/2015 | Non-infected | Commercial kit |
| AC141-7 | Cruzeiro do Sul | 04/20/2015 | Non-infected | Commercial kit |
| AC142-1* | Cruzeiro do Sul | 04/20/2015 | Non-infected | Commercial kit |
| AC143-7 | Cruzeiro do Sul | 04/21/2015 | Infected | Commercial kit |
| AC143-9 | Cruzeiro do Sul | 04/21/2015 | Non-infected | Commercial kit |
| AC144-14* | Cruzeiro do Sul | 04/21/2015 | Infected | Commercial kit |
| AC144-15 | Cruzeiro do Sul | 04/21/2015 | Infected | Commercial kit |
| AC144-17 | Cruzeiro do Sul | 04/21/2015 | Infected | Commercial kit |
| AC144-18* | Cruzeiro do Sul | 04/21/2015 | Non-infected | Commercial kit |
| AC144-19* | Cruzeiro do Sul | 04/21/2015 | Infected | Commercial kit |
| AC144-22 | Cruzeiro do Sul | 04/21/2015 | Non-infected | Commercial kit |
| AC173-25 | Cruzeiro do Sul | 04/24/2015 | Non-infected | Commercial kit |
| AC173-27 | Cruzeiro do Sul | 04/24/2015 | Non-infected | Commercial kit |
| AC354-7** | Mâncio Lima | 06/04/2015 | Infected | Salt method |
| AC360-21 | Mâncio Lima | 06/05/2015 | Infected | Salt method |
| AM40-106 | Lábrea | 08/02/2015 | Infected | Salt method |
| RO161-24 | Machadinho D'Oeste | 10/21/2015 | Non-infected | Salt method |
| RO161-26 | Machadinho D'Oeste | 10/21/2015 | Infected | Salt method |
| RO161-30 | Machadinho D'Oeste | 10/21/2015 | Infected | Salt method |
| RO163-29 | Machadinho D'Oeste | 10/21/2015 | Infected | Salt method |
| RO165-48 | Machadinho D'Oeste | 10/21/2015 | Infected | Salt method |

*PCR failed.

**16S rRNA sequencing failed.
