# Supplementary material for: Bacterial diversity associated with the abdomens of naturally Plasmodium-infected and non-infected Nyssorhynchus darlingi
Source: BMC Microbiol. 2020 Jun 25;20:180. doi: 10.1186/s12866-020-01861-0 (PMC7315559; doi:10.1186/s12866-020-01861-0)

**Additional file 3.** Venn diagram display the number of unique bacterial OTUs in *P*-negative and *P*-positive *Ny. darlingi*. Number of OTUs after filtering non-bacterial sequences.


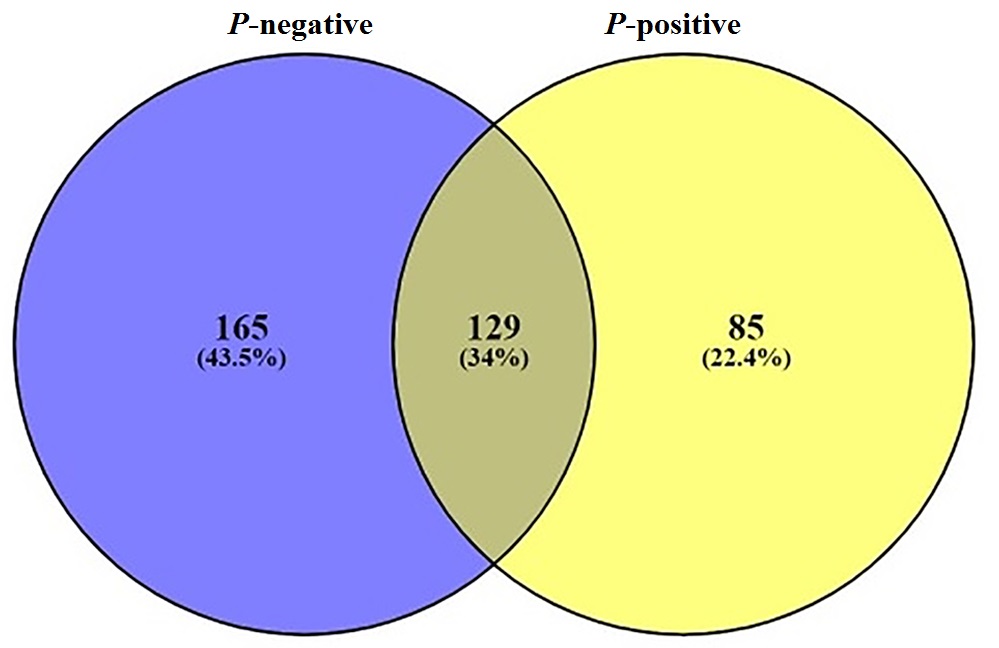

Supplement: Supplementary file 3 — Additional file 3. Venn diagram display the number of unique bacterial OTUs in P-negative and P-positive Ny. darlingi. Number of OTUs after filtering non-bacterial sequences. [file 12866_2020_1861_MOESM3_ESM.docx]
