# Supplementary material for: Bacterial diversity associated with the abdomens of naturally Plasmodium-infected and non-infected Nyssorhynchus darlingi
Source: BMC Microbiol. 2020 Jun 25;20:180. doi: 10.1186/s12866-020-01861-0 (PMC7315559; doi:10.1186/s12866-020-01861-0)

**Additional file 4**. Composition of abundant OTUs at family level of all 16 samples after filtering non-bacterial sequences. Samples collected in Cruzeiro do Sul: AC114-1, AC141-7, AC143-9, AC173-25, AC173-27, AC143-7, AC144-22, AC144-15 and AC144-17. Sample collected in Mâncio Lima: AC360-21. Sample collected in Lábrea: AM40-106. Samples collected in Machadinho D’Oeste: RO161-24, RO161-26, RO161-30, RO163-29 and RO165-48.


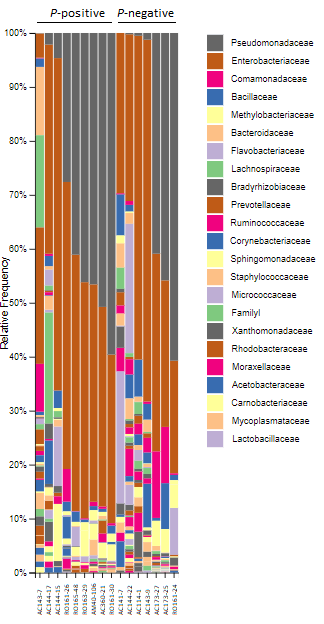

Supplement: Supplementary file 4 — Additional file 4. Composition of abundant OTUs at family level of all 16 samples after filtering non-bacterial sequences. Samples collected in Cruzeiro do Sul: AC114–1, AC141–7, AC143–9, AC173–25, AC173–27, AC143–7, AC144–22, AC144–15 and AC144–17. Sample collected in Mâncio Lima: AC360–21. Sample collected in Lábrea: AM40–106. Samples collected in Machadinho D’Oeste: RO161–24, RO161–26, RO161–30, RO163–29 and RO165–48. [file 12866_2020_1861_MOESM4_ESM.docx]
