# Supplementary material for: Bacterial diversity associated with the abdomens of naturally Plasmodium-infected and non-infected Nyssorhynchus darlingi
Source: BMC Microbiol. 2020 Jun 25;20:180. doi: 10.1186/s12866-020-01861-0 (PMC7315559; doi:10.1186/s12866-020-01861-0)

**Additional file 5.** Rarefaction curves of OTUs (97% similarity) of V4 region of 16S rRNA gene sequences from 16 samples. Rarefaction curves were generated in Qiime2 (2019.1 version). Bars correspond to the standard deviation in each depth step after 10 iterations (rarefied tables computed at each sampling depth).


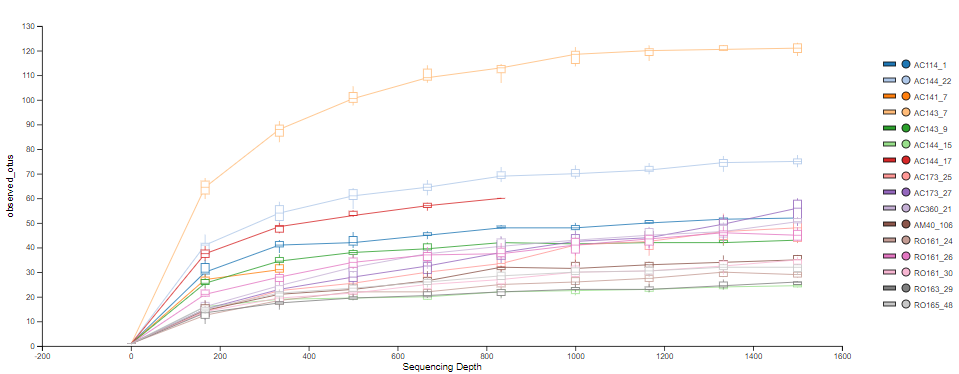

Supplement: Supplementary file 5 — Additional file 5. Rarefaction curves of OTUs (97% similarity) of V4 region of 16S rRNA gene sequences from 16 samples. Rarefaction curves were generated in Qiime2 (2019.1 version). Bars correspond to the standard deviation in each depth step after 10 iterations (rarefied tables computed at each sampling depth). [file 12866_2020_1861_MOESM5_ESM.docx]
