# Supplementary material for: Bacterial diversity associated with the abdomens of naturally Plasmodium-infected and non-infected Nyssorhynchus darlingi
Source: BMC Microbiol. 2020 Jun 25;20:180. doi: 10.1186/s12866-020-01861-0 (PMC7315559; doi:10.1186/s12866-020-01861-0)
Supplement: Supplementary file 6 — Additional file 6 Box plot of Shannon index for P-negative and P-positive groups. Center lines show the medians; box limits indicate the 25th and 75th percentiles; whiskers extend to 5th and 95th percentiles, outliers are represented by dots. [file 12866_2020_1861_MOESM6_ESM.docx]

**
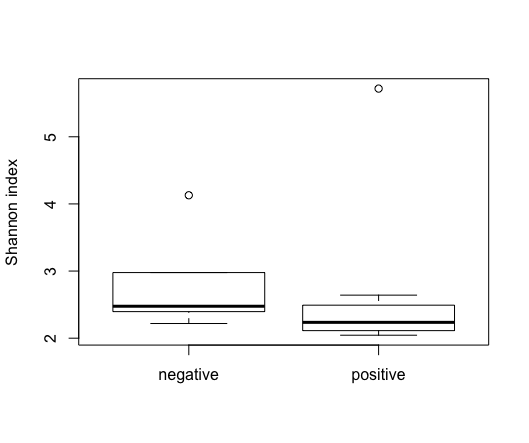
Additional file 6.** Box plot of Shannon index for *P*-negative and *P*-positive groups. Center lines show the medians; box limits indicate the 25th and 75th percentiles; whiskers extend to 5th and 95th percentiles, outliers are represented by dots.
