# Supplementary material for: Bacterial diversity associated with the abdomens of naturally Plasmodium-infected and non-infected Nyssorhynchus darlingi
Source: BMC Microbiol. 2020 Jun 25;20:180. doi: 10.1186/s12866-020-01861-0 (PMC7315559; doi:10.1186/s12866-020-01861-0)
Supplement: Supplementary file 7 — Additional file 7. Sequence of the oligonucleotides used to amplify the V4 region of the 16S rRNA gene. [file 12866_2020_1861_MOESM7_ESM.docx]

**Additional file 7.** Sequence of the oligonucleotides used to amplify the V4 region of the 16S rRNA gene.

|  | Sequences (5' - 3') |
| --- | --- |
| Forward | ATGATACGGCGACCACCGAGATCTACACTATGGTAATTGT GTGCCAGCMGCCGCGGTAA |
| Reverse | CAAGCAGAAGACGGCATACGAGAT ************ AGTCAGTCAGCC GGACTACHVGGGTWTCTAAT |

* Barcode sequence.
